# Setting and Reaching Targets with Computer-Assisted Cochlear Implant Fitting

**DOI:** 10.1155/2014/646590

**Published:** 2014-03-16

**Authors:** Bart Vaerenberg, Geert De Ceulaer, Zoltán Szlávik, Patrizia Mancini, Andreas Buechner, Paul J. Govaerts

**Affiliations:** ^1^The Eargroup, Herentalsebaan 75, B-2100 Antwerp-Deurne, Belgium; ^2^Laboratory of Biomedical Physics, University of Antwerp, Belgium; ^3^Department of Computer Science, VU University Amsterdam, The Netherlands; ^4^Department of Sense Organs, University Sapienza, Rome, Italy; ^5^Department Otolaryngol, Medizinische Hochschule Hannover, Germany

## Abstract

*Objective*. The paper aims to demonstrate the feasibility of defining a substantial set of psychoacoustic outcome measures with preset targets and to adopt a systematic methodology for reaching these targets in a large group of subjects, by more than one clinical centre. *Design*. Retrospective data analysis. *Setting*. Multicentre with 14 participating centres. *Patients*. 255 adults and children using the Advanced Bionics HiRes90k cochlear implant. *Intervention*. Target driven fitting with the fitting to outcomes expert (FOX) system. *Main Outcome Measures*. For each patient, 66 measurable psychoacoustical outcomes were recorded several times after cochlear implantation: free field audiometry (6 measures) and speech audiometry (4), spectral discrimination (20), and loudness growth (36), defined from the A§E test battery. These outcomes were reduced to 22 summary variables. The initial results were compared with the latest results. *Results*. The state of the fitting process could be well monitored by means of the measured variables. The use of the FOX computer assisted CI-programming significantly improved the proportion of the 22 variables on target. When recipients used the automated MAPs provided at switch-on, more than half (57%) of the 22 targets were already achieved before any further optimisation took place. Once the FOX system was applied there was a significant 24% (*P* < 0.001) increase in the number of targets achieved. *Conclusions*. This study demonstrates that it is feasible to set targets and to report on the effectiveness of a fitting strategy in terms of these targets. FOX provides an effective tool for achieving a systematic approach to programming, allowing for better optimisation of recipients' MAPs. The setting of well-defined outcome targets allowed a range of different centres to successfully apply a systematic methodology to monitoring the quality of the programming provided.

## 1. Introduction

Cochlear implants (CI) have become the standard treatment for bilateral severe to profound hearing loss with over 30,000 recipients implanted per year worldwide. Cochlear implant (CI) processors must be appropriately programmed and customized for the recipient [1, 2]. The aim of this is to set a number of parameters to ensure that the electrical pattern generated by the device in response to sound, yields optimal speech intelligibility. Several electrical parameters are available and all their values together are commonly called the MAP. Finding and programming the optimal values for a recipient is commonly called the act of fitting. It is achieved using proprietary software and a hardware interface connected to the processor, and depends on behavioral responses from the CI recipient.

We've recently conducted a global survey to make an inventory of the current practice in CI fitting worldwide [3]. Data were obtained from 47 centres from 17 different countries and 5 different continents. The analysis was based on a written questionnaire, a cross-sectional analysis of 5 consecutive fitting sessions for each centre, a 2-day group debate, and a 2-hour individual oral interview with each centre. It was concluded that current clinical practice in most centres could be defined as setting global profiles of maximum current levels and to a lesser extent of minimum current levels, mainly based on subjective loudness perception by the CI user. Other MAP parameters were rarely modified. It was also shown that measurable targets were only defined for pure tone audiometry. Huge variation appeared to exist across centres in virtually all aspects of CI fitting. The authors concluded that in the absence of targets or well defined outcome measures, it is impossible to compare all these differences or to judge whether some yield better results or are more efficient than others.

Hence, the authors believe that optimizing the process of CI fitting requires defining outcome measures and targets and adopting systematic approaches and algorithms to reach target. At present, there are no agreed standards or targets for both what should be adjusted, or the outcomes expected. Subjective loudness or other comfort measures are relevant, but it should be taken for granted that professionals in the field are aware of this and take care of this. Comfort as such can hardly suffice as target for such an intrusive and costly intervention as cochlear implantation. Placing an implant in the cochlea aims at taking over the function of this sensory organ and it seems obvious that any target should relate to a functional aspect of this organ. This function is the coding of sound and many features of this are well known. Psychoacoustic tests aim at testing the coding of these features in the clinic. Sound field audiograms provide a measure for the correct setting of MAP parameters and targets of 30 dB HL are used by many centers [3–5]. But audiometric thresholds only partially reflect cochlear performance. The core function of the cochlea is to code for the differences in intensity and spectral content. Assessing this requires supraliminal tests. Speech perception measures are often used but results do not depend on a good cochlear functioning alone, but also on central processing of sound and cognitive capacities. Irrespective of the speech material used, results on speech perception tests in the CI population typically range between 0 and 100% and the factors identified so far merely explain a few percentages of the variation between CI recipients [6]. Therefore, it is very difficult to define preset speech audiometrical targets for individual CI recipients.

The Eargroup decided many years ago to use a fixed set of outcome measures to assess the state of the aided cochlea after implantation; this set of tests consists of tonal audiometry, speech audiometry, and two tests of the A§E psychoacoustical test battery (Otoconsult, Antwerp, Belgium), namely, the spectral discrimination and loudness scaling tests [7]. This provides a method of continuously monitoring the “auditory state” of a CI recipient over time and goes beyond the level of subjective feedback alone. The use of the test battery also provides a set of measurable targets, which assess the auditory system at psychoacoustic level and can be compared to normal values. For each of the measured points in this test battery we defined targets for the performance level considered acceptable (see material and methods Table 2 and discussion). These targets are near to the normal values as found in hearing subjects. If the target is not reached, then performance is considered suboptimal and changes to the MAP may be indicated.

The software application Fitting to Outcomes eXpert (FOX) system, described in previous papers, introduced a systematic methodology to make adjustments to the MAP, based on the target outcomes from the A§E test battery [8, 9]. FOX is a software tool that uses a deterministic logic, based on a set of preprogrammed rules, to recommend changes to a MAP to improve outcome. The recommendations are presented to the audiologist who remains in charge and has the option to either accept or overrule the advice. The outcome measures are then repeated and used to determine if a parameter change has been effective in improving performance. A particular feature of FOX is the use of 10 incremental auto MAPs for the initial period of adaptation after switch on. This approach to predefined MAP settings in the early stages has also been used by others, but based on eCAP measures recorded intraoperatively [10]. The FOX MAPs however, are based on statistical analysis of all the ideal or “green” MAPs on the database, defined as MAPs where recipients have reached the target outcomes [9].

The purpose of this study is to demonstrate the concept and feasibility of process optimisation by setting targets in a substantial set of psychoacoustic outcome measures and adopting a systematic methodology for reaching these preset targets in a large group of subjects, by more than one clinical centre.

## 2. Methods

A retrospective study was conducted to assess the results of computer assisted CI fitting in terms of a set of psychoacoustic outcome measures.

### 2.1. Subjects

The data for 255 consecutive subjects fitted, almost all (*N* = 228) from switch-on, with the FOX programming system from January 2008 were retrospectively extracted from the FOX database. All subjects used an Advanced Bionics (AB) HiRes90k device (Advanced Bionics LLC, USA), as FOX was until recently only set up for use with AB software. The CI recipients came from 14 different centres all of whom followed the same procedure. Most came from the Eargroup in Antwerp, Belgium (152), four centres contributed at least 10 subjects (21 each from the University Sapienza in Rome, Italy and from the MHH University in Hannover, Germany, 17 from the Yorkshire Cochlear Implant Service in Bradford, UK and 10 from the University Hospital in Nijmegen, the Netherlands) and 10 centres (see acknowledgments) from France, India, Italy, Lebanon, Morocco, and the UK contributed between one and nine CI recipients each.

### 2.2. Fitting Procedure

All the CI recipients were fitted by an experienced audiologist who was assisted by FOX according to the procedures outlined in Govaerts et al. [8] and Vaerenberg et al. [9]. Briefly, the recipient received the first statistically derived auto MAP at switch-on, with T and M levels set to approximately 20 and 90 clinical units respectively, T-mic only selected and volume range set to ±5%. The recipient was then instructed to move stepwise to each of the next maps every second or third day and to try and move up to auto MAP 5 or higher, but to stop as soon as it becomes uncomfortable. This typically took two weeks. Once this level was reached, the fine-tuning of the MAP assisted by FOX began. This was done in a staged procedure comprising three sessions over three months (Table 1). Targets were defined for all tests from the psychoacoustic test battery and are listed below. The initial focus was on detection and discrimination of the acoustic signal, using audiometry and A§E phoneme discrimination as outcome measures. Thereafter identification was optimised using loudness scaling and speech audiometry. If the measured outcome was within the target range defined, the audiologist (assisted by FOX) did not undertake any modifications. If the outcome was not within target, FOX made recommendations for modifying the MAP in an attempt to bring the outcome closer to target. In most of the cases, the audiologist accepted the recommendations made, although he/she had the option to overrule them. The same outcome was then measured again and if still out of target FOX made further suggestions, changing the MAP several times before resting its case.

### 2.3. Outcome Measures and Targets

The following outcome measures were used to assess the results.

*Free Field Audiometry (6 Raw Data Points).* Thresholds determined in Free Field with loudspeaker positioned at 1 m from the subject and warble tones presented at 250, 500, 1000, 2000, 4000, and 8000 Hz.

*Spectral Discrimination (20 Raw Data Points)*. A§E phoneme discrimination using 20 speech sound contrasts (a-r, u-⎰, u-a, u-i, i-a, o-a, i-*ε*, m-z, s-⎰, *ε*-a, u-o, *ə*-a, *ə*-o, *ə*-*ε*, *ə*-I, z-s, v-z, *ə*-u, u-y, y-i) presented at 70 dB SPL in an oddity paradigm, 1 m from the subject (see [7] for test details). A result of yes or no was recorded for the discrimination of each contrast, yielding 20 results, which were grouped to one variable representing the cumulative score on 20.

*Loudness Growth Function (36 Raw Data Points).* A§E loudness scaling test using 1/3rd octave narrow band noises, centred at 250, 1000, and 4000 Hz. A 1876 ms stimulus was presented twice at each level and scored on a visual analogue scale ranging from 0 (inaudible) to 6 (too loud). Levels were randomly presented at 5 dB increments between 30 and 80 dB HL. This yielded 36 values. Scores were pooled for four different levels (30–35–40 dB HL, 45–50–55 dB HL, 60–65–70 dB HL and 75–80–85 dB HL), leading to 12 variables for further analysis.

*Speech Audiometry (4 Raw Data Points).* Monosyllabic CVC word lists with phoneme scoring presented at 40, 55, 70, and 85 dB SPL, 1 m from the subject. The slope between two neighbouring points was then calculated, yielding 3 variables for further analysis.

This yielded 66 raw data points, some of which were grouped such that the final number was reduced to 22 outcome variables (listed in Table 2) for further analysis. Audiometry was performed in all subjects, but the other tests were not performed in all because, due to age or cognitive ability, this was not always possible. Table 2 shows how many patients underwent each outcome measure at least twice during their follow up.

For each of these 22 outcome variables, a target and near target for acceptable performance was defined as shown in Table 2. The rationale for these targets is addressed in the discussion section. Briefly, the targets for audiometry were 30 dB, which corresponds to the lower limits of the device microphone and the front end technology. The targets for spectral discrimination were set at 85%, since this is a prerequisite for good speech understanding. The targets for loudness scaling were the 95% confidence interval in hearing subjects and the targets for the speech audiometric slopes were set empirically at ±15%. We calculated two measures for success: (1) the target hit rate (THR) for each outcome variable and (2) the subject's hit rate (SHR) for each CI recipient. This was done at two moments, namely, after switch-on (initially) and when the optimisation was considered to be completed by FOX (finally). The THR was calculated for each outcome measure as the percentage of subjects who had reached the target. If the target was not reached we looked at the time interval between the initial and the final measurement. A small interval indicates that the fitting process may not yet have been finished and that further optimisation might still be possible if additional programming sessions would be undertaken. The THR in that case might be an underestimation of the real success rate. The SHR was calculated for each subject as the percentage of the 22 targets which was reached by the subject. In addition for both THR and SHR we also calculated the percentages with results within the “almost on target” range according to the definitions of Table 2. These will be referred to as tolerant THR (tTHR) and tolerant SHR (tSHR) henceforth.

Descriptive statistics were used to present the results as histograms for THRs and box and whisker plots for SHRs. Nonparametric statistics were used to compare the initial and final THRs and SHRs (Wilcoxon paired rank tests) with a cut-off level of significance set at 0.05.

## 3. Results

Sixty-six psychoacoustic points were measured to monitor the fitting in 255 consecutive CI recipients. Some results were grouped such that a total of 22 outcome variables were obtained to describe the “state” of the CI fitting process. For all variables a target was defined in a strict sense (on target) and a more tolerant sense (almost on target). Hence the state of the process was measured at two moments, marked as initial and final. The initial state refers to the first time that the outcome was measured, which is typically after the automated switch-on procedure. It therefore reflects the success rate of this start-up procedure. The final state is the last time the outcome was measured. Since all CI-recipients were fitted for target (by the audiologist assisted by FOX), this final state reflects the success rate of this fitting approach.

**Figure 1:**
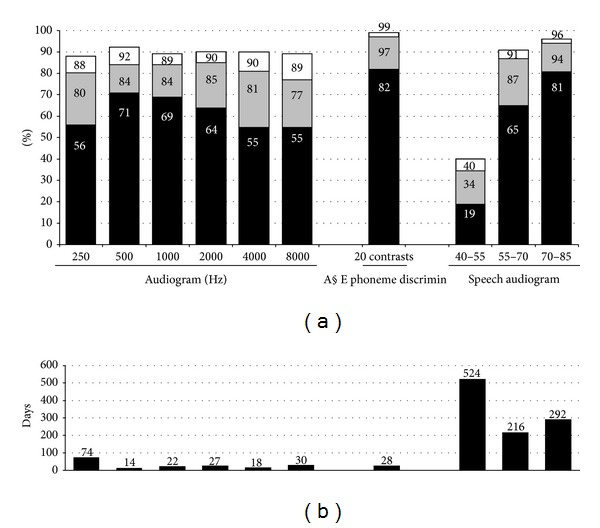
(a) shows the percentages of CI-users who performed on target (THR) at initial testing (black), final testing (gray), and almost on target (tTHR) at final testing (white) on Audiometry, A§E phoneme discrimination (A§E phoneme discrimin), and Speech Audiometry. Part (b) shows the interval between the initial and final measurement for those CI-users who did not reach target at the latest measurement.

**Figure 2:**
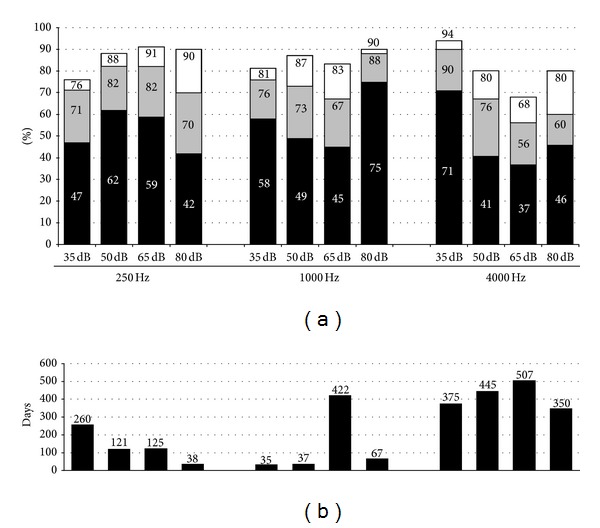
The figure shows the results for Loudness Scaling at 250, 1000, and 4000 Hz. See Figure 1 for interpretation.

The THRs and tTHRs of all 22 outcome variables individually are shown in Figures 1(a) and 2(a) and also in Table 2. After the initial switch-on procedure, the THR ranged from 19% for the variable (Speech Audiometry 40–55 dB) to 82% for the variable (Spectral Discrimination). After this switch-on procedure the computer (FOX) assisted fitting improved the THR for all of the individual outcome variables (median improvement = 21%; *P* < 0.001). As displayed by the figures, the THR was substantially different across outcome variables. For instance, the loudness scaling results show that the coding of soft sounds at 4000 Hz already reached target at initial evaluation in 71% of the subjects and that this improved to 90% at the final evaluation (Figure 2(a)). This is in contrast to the speech audiometry at soft presentation levels where the slope between the 40 and 55 dB SPL presentation levels only reached target in 19% of the subjects at the initial evaluation and in 34% at the final evaluation (Figure 1(a)). In both cases, the subjects who did not reach target had been evaluated more than a year after the initial stage (375 days for the loudness scaling and 524 days for the speech audiometry, Figures 2(b) and 1(b), resp.). From this it can be inferred that sufficient time had passed to try optimizing these outcomes and that it would be unlikely that they would further improve.

**Figure 3:**
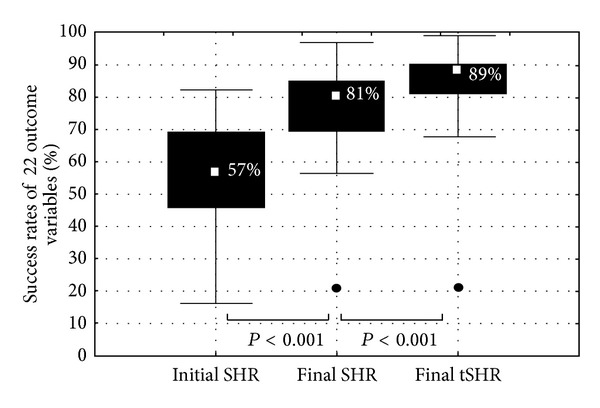
The figure shows the distribution of the success rates of 22 outcome variables (SHR) with the median value (central dot), quartile range (box) and range (whiskers).

The SHR results are shown in Figure 3. This representation allows analyzing how close CI recipients come to target when all 22 measures are taken into consideration. For instance, it shows an average SHR of 57% after switch-on by means of the automated MAPs and before any further optimisation took place. This means that the average CI recipient is on target for almost 13 of the 22 outcome variables. The computer-assisted fitting yielded a significant improvement of 24% in SHR, from 57% to 81% (*P* < 0.001). A further significant improvement of 8% (*P* < 0.001) was seen when the almost on target values were applied.

## 4. Discussion

Optimizing any process requires (1) a number of parameters to be adjusted within specific constraints, (2) quantitative or objective performance measures that need to reach predefined targets, and (3) a systematic approach with methods and algorithms rather than trial and error. When applying this to the process of CI fitting, the first requirement refers to the MAP parameters which can be modified by means of the CI programming software. The next 2 requirements are not obvious, as revealed by the global survey which has recently been conducted [3]. At present CI fitting is performed by experts in the field who have an idea of what the expected level of performance for an individual recipient should be and who make MAP adjustments if this target is not reached. Assessing the success of changing a parameter usually relies solely on patient feedback. There is no universal set of quantitative measures which is commonly used to quantify the auditory state of the aided cochlea, and for which well-defined targets are commonly accepted. Also, the basis for adjusting the MAP parameters is often heuristics or trial and error. Systematic approaches are lacking both in textbooks [2, 11] and as revealed by the global survey [3].

This report shows that the setting of well-defined outcome targets did allow a range of different centres to apply a systematic methodology to monitoring the quality of the programming provided. In an age where good clinical practice requires an evidence based approach, it is essential to have the ability to objectively monitor and audit the success of the treatment provided. The use of clear targets enables audiologists to define what is meant by an optimised MAP and provides consistency across different professionals and centres.

For outcomes to be effective they must be measureable in most clinical settings and reliably repeatable. They must also provide an accurate assessment of auditory performance and preferably be independent of the language spoken. The auditory system is complex and therefore requires a complex assessment system; one single measure is unlikely to be sufficient to provide all the information required. Like any other sensory organ, the cochlea is responsible for detecting its particular signal, sound, and for discriminating two sounds which differ in one of their components. In the absence of a global consensus on such targets, we have chosen the psychoacoustical targets as used in this study because we believe that, within the context of programming, they reflect well the state of the aided cochlea. They combine measures at the level of detection (audiometry), spectral discrimination (A§E phoneme discrimination) and identification (A§E loudness scaling and speech audiometry). They cover the coding of the sound features intensity and spectral content and most can be used for both adults and children, as they do not require a high cognitive or language level and are easy to implement across a wide range of centres. One can argue the choice of outcome variables and with this paper we do not intend to state that the variables chosen here compose the best selection. We do intend to open the debate and to make the point that a consensus would be very helpful in moving forward the discussion on the quality of CI fitting. The targets and “tolerant” targets set for each outcome were empirical though educated choices. The audiometrical targets were set at 30 db HL since this is close to the technological limit of the current CI devices, which is defined by a combination of the microphone sensitivity, the front end preprocessing and filter bank steps and the internal noise of the electronic circuitry [12]. The target for spectral discrimination was set at 85%, which means that 17 out of the 20 contrasts presented are well discriminated by the CI recipient. Previous unpublished results of our team have shown that good speech intelligibility (≥60% phoneme score on monosyllabic speech lists) is only obtained in listeners with at least 85% score on the phoneme discrimination test. The target for loudness scaling was set to be the 95% confidence interval in hearing listeners. And, for speech audiometry, we have chosen not to use absolute scores as target because there is no such value which is valid for all CI recipients. This is because speech audiometry results depend on much more than just a good replacement of the cochlear function, which explains the huge variability in this outcome across CI users [6]. On the other hand, a valid target can be to maintain the best score for a given CI user across a wide range of presentation levels. Therefore we use to present speech not at one single presentation level but rather at different levels (40–55–70–85 dB SPL) and our target is to have scores at these levels which are as close as possible to the best of all four scores. This is reflected in the slopes of the three lines connecting the four scores being as close as possible to zero; hence, we set the slopes of 0 ± 15 as empirical target.

Once these targets were set, we introduced a systematic approach to change the MAPs based on the outcome obtained. The FOX computer assisted programming system provides such a systematic approach across centres. All the centres included were able to use the FOX system effectively and to perform the tests required. In this study, the use of the FOX system significantly improved the audiologists' ability to achieve the target outcomes set at the beginning of the study. The initial switch-on MAPs provided were solely based on the statistical derivation of T, M and Gain levels, with incremental increases in T and M levels applied as the MAP number used was increased. With these MAPs, more than half (57%) of the 22 targets were already achieved before any further optimisation took place. Once the FOX system was applied and optimisation began, there was a significant 24% increase in the number of target achieved, as measured at the last fitting session. Also, the spread of SHR across subjects decreased from 66% initially (range 16–82%) to 41% finally (range 56–97%) or even 31% if near to target scores are also tolerated (tSHR range 68–99%). This indicated that the approach under study is capable of delivering robust results across different CI recipients from different CI centres.

The approach of systematic fitting for target also allows looking at and interpreting the individual results for each outcome measure (THR). For instance, FOX was able to improve the THR for free field audiometry outcomes by a minimum of 13% and by as much as 26% at 4000 Hz and 24% at 250 Hz. Although this measure merely reflects the front-end technological capacity of the device, it is remarkable to see that it still requires customized programming to achieve good results in every individual. The results for phoneme discrimination were good without any optimisation, with 82% achieving target. This is in line with previous reports on the use of FOX [9]. It is not unexpected, because cochlear implants are conceived primarily to restore the tonotopical organisation and also because the contrasts used in the A§E phoneme discrimination task are rather easy, thus causing ceiling effects. Nevertheless it is important to achieve good results on this task because spectral discrimination is one of the core tasks of the cochlea and a prerequisite for good central processing and identification. At the same time they are not sufficient to guarantee good supraliminal identification, which is reflected by the fact that for the identification tasks (loudness scaling and speech intelligibility), the THR with the Auto MAPs is considerably lower, namely, between 37% and 71%.

For speech audiometry equivalent performance across presentation levels is considered to be an area where correct fitting of the device can directly impact performance. For the un-optimised Auto MAPs, at what could be considered to be the key intensity comparison of 55 and 70 dB levels, the THR with Auto MAPs was 65%. This was improved with FOX optimisation to 87%. However, the THR for the 40 and 55 dB comparison remained low at 40%, even after optimisation. This is in line with previous reports showing that speech intelligibility at these quiet levels is very challenging [13].

The loudness scaling targets were harder to achieve with the Auto MAPs, with the 4000 Hz frequencies being the most difficult. This was again also shown in the smaller sample reported by Vaerenberg et al. [9] and it was assumed that the difficulty in measuring the loudness outcomes relates to the distorted loudness picture that some recipients have become used to during long periods of hearing aid use [9]. However, despite the difficulties, once FOX optimisation was applied, 9 out of the 12 measures showed a THR between 70–90% which represented an improvement of between 13% and 28% over the un-optimised MAPs. Again, once the “almost on target” outcomes were applied, two out of the other three measures below the 70% on target value, increased to at least 80%.

Results were based on the last available measurement and not the measurement when the fitting was considered to be optimal. Typically, a reasonable interval to achieve optimum would be around one month for audiometry and spectral discrimination and six months for loudness scaling and speech audiometry. Therefore, for some measures, the interval between the initial and final measurements was much less than that ideally required and if optimisation was continued, then further improvements in the percentage of subjects achieving target could be expected.

A final consideration is on the validity of the outcome variables chosen. Although the authors feel that the main justification for the current selection of outcome variables lies in the fundamentals of sound coding by the cochlea, as argued above, the ultimate proof of their validity will come from better speech understanding in quiet and in noise. It is beyond the scope of this paper to further address this issue, but two studies have been conducted in other centres than the Eargroup where speech understanding in quiet and in noise has been analyzed after conventional fitting compared to computer assisted and target driven fitting. The first study was conducted at MHH (Hannover, Germany), where 10 long term CI users who had always been fitted in the conventional way entered a single FOX iteration based on the 66 measured outcome points (Buechner et al., submitted). Speech audiometry with monosyllabic words in quiet improved instantaneously in 7 CI recipients and deteriorated in 3. Speech audiometry with sentences in noise improved instantaneously in 6 CI recipients and deteriorated in 4. Another Sentence Test with Adaptive Randomized Roving levels (STARR test) [14, 15] yielded better results in 4 CI recipients and worse results in 6. Although, not statistically significant, this shows that a single iteration with the computer assisted and target driven approach can further improve the speech understanding in more than 50% of CI users who have been fitted throughout many sessions by expert hands. The second study was a multicenter study conducted in 6 CI centres in Germany, France and the UK (Battmer et al., submitted). New CI recipients were randomized to enter either a conventional fitting arm or a computer assisted, target driven arm during 3 months starting at switch-on. The computer assisted, target driven approach used the 66 outcome measures as mentioned before and the FOX application to assist the audiologist. Speech audiometry was assessed in quiet and in noise at 6 months and it showed significantly better results in the computer assisted, target driven arm compared to the conventional arm both in quiet and in noise. These results, although preliminary and in small numbers, indicate that the target driven systematic approach may be considered promising.

## 5. Conclusions

This study demonstrates that it is feasible to set targets and to report on the effectiveness of a fitting strategy in terms of these targets. This is demonstrated with the FOX-assisted strategy as example. The psychoacoustical measures chosen here were selected because they measure the behavioural response to acoustic stimulation, both in terms of loudness and frequency, and provide the building blocks for eventual speech perception and language development. This study also demonstrates that the application of the FOX system provides an effective tool for achieving a systematic approach to programming, allowing for better optimisation of the MAPs, when measured by the set targets. When recipients used the automated MAPs provided at switch-on, more than half (57%) of the 22 targets were already achieved before any further optimisation took place. Once the FOX system was applied there was a significant 24% increase in the number of targets achieved. The setting of well-defined outcome targets allowed a range of different centres to successfully apply a systematic methodology to monitoring the quality of the programming provided.

## Figures and Tables

**Table 1:** Overview of the fitting procedure.

Session	Programming	Outcome measure
Switch-on	Auto MAPs loaded	None
Session 2 (2 weeks)	Electrode deactivation (if required)	Impedance telemetry, free field audiometry
Session 3 (4 weeks)	MAP optimization as recommended by FOX, but only if targets not reached	Free Field Audiometry, Phoneme Discrimination
Session 4 (10–12 weeks)	MAP optimization as recommended by FOX, but only if targets not reached	Loudness scaling, speech audiometry

**Table 2:** Overview of outcome variables with value definitions for target and close to target.

Audiological test	*N*	Outcome variable	Target	Almost on target	% on target at first	% on target at last	% almost on target at last
Audiometry	255	250 Hz	≤35 dB HL	≤40 dB HL	56	80	88
	255	500 Hz	≤30 dB HL	≤40 dB HL	71	84	92
	255	1000 Hz	≤30 dB HL	≤40 dB HL	69	84	89
	255	2000 Hz	≤30 dB HL	≤40 dB HL	64	85	90
	255	4000 Hz	≤30 dB HL	≤40 dB HL	55	81	90
	255	8000 Hz	≤30 dB HL	≤40 dB HL	55	77	89

Spectral discrimination	102	Set of 20 contrasts	≥18/20	≥17/20	82	97	99

Loudness scaling*	177	250 Hz (30–40 dB SPL)	1.1–2.8	0.8–3.1	47	71	76
	178	250 Hz (45–55 dB SPL)	1.9–3.6	1.6–3.9	62	82	88
	180	250 Hz (60–70 dB SPL)	2.9–4.4	2.6–4.7	59	82	91
	182	250 Hz (75–85 dB SPL)	4.1–5.8	3.7–6.1	42	70	90
	180	1000 Hz (30–40 dB SPL)	1.2–2.3	0.9–2.6	58	76	81
	180	1000 Hz (45–55 dB SPL)	1.9–2.9	1.6–3.2	49	73	87
	181	1000 Hz (60–70 dB SPL)	2.7–3.7	2.4–4.0	45	67	83
	182	1000 Hz (75–85 dB SPL)	3.4–5.1	3.1–5.4	75	88	90
	178	4000 Hz (30–40 dB SPL)	0.6–2.1	0.3–2.4	71	90	94
	180	4000 Hz (45–55 dB SPL)	1.3–2.7	1.0–2.4	41	67	80
	137	4000 Hz (60–70 dB SPL)	1.9–3.4	1.6–3.7	37	56	68
	178	4000 Hz (75–85 dB SPL)	2.6–4.2	2.3–4.5	46	60	80

Speech audiometry	58	Differential scores at 40 versus 55 dB SPL	−15–15%	−20–20%	19	34	40
	92	Differential scores at 55 versus 70 dB SPL	−15–15%	−20–20%	65	87	91
	89	Differential scores at 70 versus 85 dB SPL	−15–15%	−20–20%	81	94	96

*N*: number of included records; Outcome variable: see text for more information; Target dimensions: for Audiometry: dB HL; for Spectral discrimination: score on 20; for Loudness scling: average score on visual-analog scale; for speech audiometry: difference in phoneme score between 2 presentation levels (see text for details). *for loudness scaling, the target values correspond to the 95% confidence interval in hearing subjects.
